# Motherhood, Psychological Risks, and Resources in Relation to Alcohol Use Disorder: Are There Differences between Black and White Women?

**DOI:** 10.1155/2014/437080

**Published:** 2014-04-19

**Authors:** Sundari Balan, Gregory Widner, Hsing-Jung Chen, Darrell Hudson, Sarah Gehlert, Rumi Kato Price

**Affiliations:** ^1^Department of Psychiatry, Washington University School of Medicine, Medical Box 8134, 660 South Euclid Avenue, St. Louis, MO 63110, USA; ^2^Graduate Institute of Social Work, National Taiwan Normal University, Jheng-Pu Building, 5F, No. 162, Section 1, Heping East Road, Taipei City 10610, Taiwan; ^3^George Warren Brown School of Social Work, Washington University in St. Louis, One Brookings Drive, St. Louis, MO 63130, USA

## Abstract

Rates of alcohol use disorders (AUD) are generally low among women who have ever had children (mothers) compared to women who have never had children (nonmothers), presenting a *motherhood advantage*. It is unclear if this advantage accrues to “Black” and “White” women alike. Using National Epidemiological Survey on Alcohol and Related Conditions (NESARC) wave 2 cross-sectional data that is rich in alcohol use and psychological measures, we examined the following: (a) if motherhood is protective for past-year AUD among Black (*N* = 4,133) and White women (*N* = 11,017); (b) potential explanatory psychological mechanisms; and (c) the role of race. Prevalence of a past-year DSM-IV AUD was lower among White mothers compared to White nonmothers, but this same advantage was not observed for Black women. Perceived stress was a risk for all women, but race-ethnic segregated social networks and perceived discrimination predicted current AUD for Black mothers. Unlike White mothers, current psychological factors but not family history of alcohol problems predicted AUD for Black mothers. Future prospective studies should address the mechanisms by which race, motherhood, and psychological factors interactively affect AUD in women.

## 1. Introduction

Existing epidemiological evidence for alcohol use disorder (AUD) supports the notion that women who have ever had children (mothers) experience a* motherhood advantage*; that is, they exhibit lower rates of AUD than women who have never had children (nonmothers) [1–3]. A lifetime motherhood role provides a number of protections including becoming more careful with alcohol use and not having a lot of extra time to drink [3]. However, to date, no studies have examined if this advantage differs by race. The purpose of this study is to use cross-sectional national survey data to compare AUD among mothers and nonmothers, while at the same time disentangling any racial disparities. Additionally, we examine associations with individual-difference variables to illuminate potential general (e.g., perceived stress, social support, and discrimination) and race-related psychological factors (e.g., racial networks) that might explain different observed patterns by race [3–5].

In the biomedical literature, AUD is a condition impacted by genetic [6], metabolic (e.g., [7]), family history [8], and social contextual factors related to availability of alcohol [9] and access to services [10]. In the general-population epidemiological literature, the prevalence of AUD is considerably lower among women than men, which has been attributed to less access to alcohol and the different metabolic processes of women (e.g., [9]). Perhaps because of lower rates of AUD, gender specific life role patterns have been underexplored [11]. Family history of an alcohol problem presents a significant risk despite the fact that the risk is lower for women than men [8]. Cultural norms and family processes are more significant for women than for men [12].

Racial disparities in the lifetime prevalence of AUD are also well recognized in the literature. Lifetime prevalence of AUD is lower among Black women as compared to White women [11]. Although the incidence of AUD is lower among Black women when compared with White women, it is more likely to be* chronic* among Black women as compared to White women [13]. That is to say that it persists through several life role transitions including marriage and through changing employment. To date, the role of motherhood for AUD has largely been neglected. Focusing on the effects of motherhood specific to Black women is significant because of the evidence that Black women have experienced high levels of chronic stress which has been associated with high rates of disease [14]. This chronic stress appears to manifest as a lifelong process with the worst outcomes experienced by Black women in their middle adulthood [14]. The* intersection* [15, 16] of race with AUD in women has not been examined in the current literature. In the current study we addressed this gap by examining the intersecting or nonadditive effects of race and motherhood.

The evidence supports a motherhood advantage for AUD. Women who have never had children have higher rates of AUD than women who were ever mothers [3]. Using a prospective epidemiological study design, Chilcoat and Breslau [2] found a protective effect of transition to parenthood for the development of AUD. Perhaps this is because women who are considering motherhood are less likely to be abusing substances in the first place [17, 18]. Second, women who are considering biological parenthood are more likely to access medical screening which would enable them to find adequate resources for treating an existing AUD [18]. Paradoxically, in the face of social disadvantage, medical screening may not be protective because women may be forced to hide their drinking problem [19]. Furthermore, poor women from racially disadvantaged groups may not have access to medical screening. Motherhood and additional role responsibilities increase perceived stress which is a risk factor for AUD. Under these circumstances, motherhood may be a source of disadvantage for some women and exacerbate racial disparities. Motherhood can increase role responsibilities [18]. Further, it can increase the burden of scarce economic or social support resources [20, 21]. Women who have experienced gender and racial discrimination in services are less likely to utilize services later [21, 22]. Among treatment seekers, mothers worried that they would lose custody of their children if they sought treatment for alcohol problems [19]. For some racial groups, social disadvantages and racial disparities are increased through the effects of discrimination [21]. Black mothers may not utilize treatment adequately or may experience considerably more disadvantages including racial discrimination.

Although transition to motherhood is generally considered protective, lifetime motherhood effects for AUD have not been considered. No study we came across examined differences between Black and White women. This is particularly significant as it relates to Black women, among whom different familial arrangements have been documented [23, 24]. Because of these differential family arrangements, existing health disparities, and experience of chronic stress [5, 14], the connections between lifetime motherhood status and AUD may exhibit different patterns among Black and White women, a point we considered in the current study.

**Figure 1:**
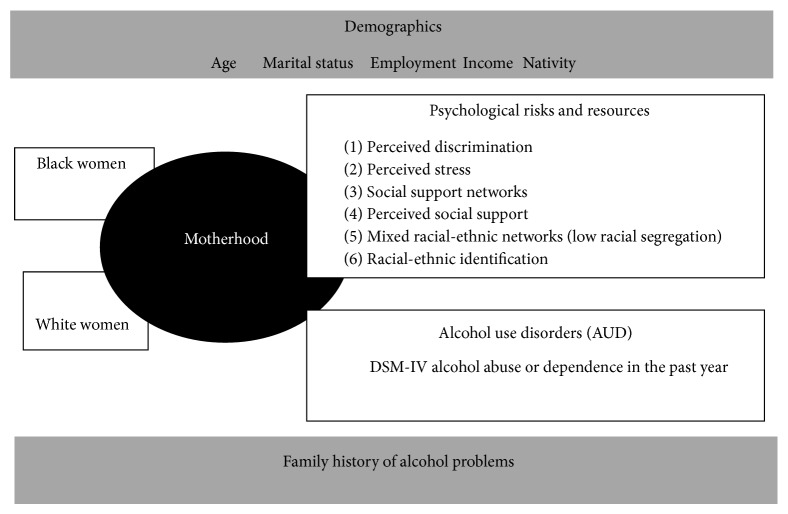
Conceptual model underlying current study of alcohol use disorders (AUD) among Black and White women.

Figure 1 presents the conceptual model for our study. The underlying psychological processes which can explain racial differences in the links between motherhood and AUD have also been neglected in the epidemiological literature. Conceivably, the effect of mother status and race may intersect and cause differences in the way risks and resources influence AUDs [15, 16, 22]. Although no study compared mothers and nonmothers by race, a growing body of literature supports the notion that there are health disparities and being Black does indeed affect health. Among the psychological mechanisms, perceived discrimination has been heavily implicated for the poor health status of Black Americans [5, 22, 25]. For both Black and White women, gender discrimination may also be an intersecting factor that increases risks for psychiatric problems in general [26] and alcohol use disorders among some minorities [27]. Although there is evidence that discrimination is related to alcohol problems among Blacks [5, 22], no study that we came across used DSM-IV AUD criteria.

Differences in how Black and White women perceive stress have been implicated as another important mechanism which explains different health outcomes experienced by Blacks. Some studies suggest that Blacks are likely to appraise their lives as stressful at rates higher than Whites [19, 28]. This is evidenced in the increased chronic stress experienced by Black women in particular when compared with White women and with men [14]. The types of social support are also important and must be considered for AUD [29]. However, the relationship between social support and AUD is currently mixed. Although the general expectation is that social support has a positive effect on AUD, some studies do suggest that social support may not be associated with AUD [30].

Race-ethnic identity and race-ethnic networks also present themselves as important psychological mechanisms that can explain racial differences in health [28, 31], but their relationships to AUD are complex [32]. They may either have a positive or negative effect on AUD. Identification with one's group in the sense that one interacts with members of the group can represent a source of instrumental support [31, 32]. Furthermore, this enables racial and ethnic group members to cope and recover from multiple everyday stressors, especially discrimination, which are known to pose increased risk for heavy drinking and alcohol abuse [25].

In epidemiological literature that uses DSM-IV alcohol use disorder criteria, psychological measures of racial networks are rare and few studies include an individual's own reported networking patterns. Instead, neighborhood measures are more widely studied. Unequivocally, these studies suggest that racial segregation exposes women to risk factors such as economic disadvantage [33–35] and increased availability of alcohol for coping [36]. Furthermore, structural factors, such as features of the built environment that foster isolation by race (e.g., residential segregation), may reinforce this effect. In one study, Black women in race-segregated neighborhoods were increasingly targeted by alcohol advertisements [36]. Isolation from other racial groups also appears to be a key stressor for Black women in many health areas [37]. Because racial networks are important factors but psychological aspects are understudied we include this measure in the current study.

Overall, intersecting motherhood and racial group differences are not sufficiently explored in the etiology of AUD. Although absence of a motherhood effect may be one reason why a chronic pattern of AUD is observed among Black women, these differences have not been examined. The psychological resources and risks which may explain any racial differences have also received insufficient attention. Using national survey data, the current study disentangles racial group differences focusing on motherhood effects. We expected a motherhood advantage to be seen for White women but perhaps not among Black women. We also expected that the groups will differ on psychological risks such as discrimination and resources such as racial networks and that Black mothers would experience the worst disadvantages.

## 2. Methods

### 2.1. Sample

We utilized a subsample from the second wave of the National Epidemiologic Survey on Alcohol and Related Conditions (NESARC) conducted in the years 2004-2005. Data for the original survey was collected using face-to-face and computer assisted personal interviews in respondents' homes, yielding a cumulative survey response rate of 81% [38]. For the current study, adult female lifetime drinkers who were self-identified as either “White” (*N* = 11,017) or “Black” (*N* = 4,133) were included. This dataset was selected because it was rich in AUD and psychological measures. Psychological measures were only available in wave 2 data and hence only this wave was used in the current analysis. In order to maintain temporal consistency in measures, only past-year alcohol use disorder and psychological conditions at the time of interview were used.

### 2.2. Measures

#### 2.2.1. Alcohol Use Disorder (AUD)

NESARC used the alcohol use disorder and associated disabilities interview schedule-IV (AUDADIS-IV: 38) to measure alcohol related factors. For the current study, we used NESARC preconstructed measure (available in wave 2 data) of alcohol abuse and/or dependence anytime during the past year. Alcohol abuse and dependence criteria corresponded to the* Diagnostic and Statistical Manual of Mental Disorders, 4th ed*., alcohol abuse and dependence disorder symptoms [39]. These included recurrent drinking resulting in failure to fulfill major role obligations; recurrent drinking in hazardous situations; recurrent drinking related legal problems; continued drinking despite recurrent social or interpersonal problems caused or exacerbated by drinking; persistent desire to drink; and continued drinking despite problems [39].

#### 2.2.2. Demographics

These included a continuous measure of current age and dichotomous measures of current marital status (single versus married/in a relationship), family income (less than USD 20,000/greater than or equal to USD 20,000), nativity (i.e., born in the United States or not), and employment status (currently employed for over 35+ hours a week or not). A single item, preconstructed measure available in NESARC wave 2 of the total number of children women “ever had,” was also included. It accounted for any children or adopted/foster/step children. Although this measure gave us lifetime motherhood status, this measure could not distinguish between biological and nonbiological parenthood. Furthermore, this measure could not account for age of children. These remain limitations of the current study.

#### 2.2.3. Major Predictors

*(i) Family History of Alcoholism*. Four items from wave 1 data were included to reflect an alcohol problem in a full parent or full sibling.

*(ii) Perceived Discrimination*. Six items measured perceived discrimination (e.g., during past year how often did you experience discrimination in ability to obtain health care or health insurance coverage because of your race/ethnicity? During past year how often did you experience discrimination in how you were treated when you got care because of your race/ethnicity? During the past year how often did you experience discrimination in public on the street, in stores or restaurants, because of your race/ethnicity?). Three items assessed perceived discrimination because of race/ethnicity and three similarly worded items assessed perceived discrimination because of gender. The NESARC scales were originally modified from Krieger [40]. Each discrimination question was measured on a scale from 0 to 4 (0)* never*, (1)* almost never*, (2)* sometimes*, (3)* fairly often*, or (4)* very often*. Consistent with some other approaches (e.g., [32]), the scores were dichotomized to indicate the presence of a discrimination experience in the past year.

*(iii) Perceived Stress*. We used the Perceived Stress Scale which contains four items (PSS4: 41). The PSS4 measures cognitively mediated emotional responses to events. Items on this scale (e.g., able to control important things in their lives) were measured as follows: (0)* never*, (1)* almost never*, (2)* sometimes*, (3)* fairly often*, and (4)* very often*. Herd and Grube [31] reported high reliability for the AUDADIS version. Two of the four items were reverse coded such that high scores indicated greater perceived stress. A mean perceived stress score was computed for each respondent in cases in which an individual responded to at least two of the four items (mean = 1.99, SD = 0.74, and range = 1–5).

*(iv) Social Support Networks*. The Social Network Index (SNI) developed by Cohen and Hoberman [41] was used to measure social networks. This measure assessed participation in 12 types of social relationships/resource including spouse, neighborhood, and community. Participants indicated whether they had any contact with any of these relationships or resources in the past two weeks. If any of these networks was active, a positive score was assigned. A summary score was accordingly computed to reflect the size of an individual's network (mean = 8.78, SD = 0.93, and range = 0–10).

*(v) Perceived Social Support*. The 12-item Interpersonal Support Evaluation List (ISEL 12 : 43) was used to measure availability of helpful social resources. Respondents reported items on a scale ranging from 1 to 4: (1)* definitely false*, (2)* probably false*, (3)* probably true*, and (4)* definitely true*. A high score was indicative of high social support (mean = 3.51, SD = 0.47, and range = 1–4).

*(vi) Mixed Racial-Ethnic Networks*. Four items assessing composition of networks were used. Each of the items addressed a particular interpersonal domain: (1) close friends; (2) party visits; and (3) visitors. Participants responded on a five-point scale ranging from 1 to 5: (1)* all from my race/ethnic group*; (2)* more from my race/ethnic group*; (3)* about half and half*; (4)* more from other race/ethnic groups*; and (5)* all from other race/ethnic groups*. Ruan et al. [42] noted high reliability of these four items. A sum of these items was obtained to compute a racial network score. Higher scores reflected mixed or nonsegregated racial networks and lower scores implied that the networks were racially segregated (mean = 6.85, SD = 2.61, and range = 1–15).

*(vii) Racial-Ethnic Identification*. This was measured by the Multigroup Ethnic Identity Measure (MEIM: 45). Participants responded to each of eight items (e.g., you have a strong sense of yourself as a member of your race/ethnic group) on a six-point rating scale ranging from (1)* strongly agree *to (6)* strongly disagree*. A mean score was obtained by summing all the values across the 8 items if at least half the items (i.e., four items) were answered by the respondent. Otherwise the measure was treated as missing (mean = 2.33, SD = 0.85, and range = 1–6).

### 2.3. Analytical Strategy

Wave 2 of NESARC used a multistage, stratified, complex sampling design in which primary sampling units (PSUs) were stratified according to certain sociodemographic criteria. Our variance estimation procedures accounted for this complex sampling design. We used wave 2 sampling weights as our analysis was restricted to wave 2 data. We used design-based analytic techniques to obtain estimates of population parameters. SAS 9.2 was used for data preparation; SAS-Callable SUDAAN version 10 [43] was used for all estimation processes because of SUDAAN's use of the Taylor series linearization estimation of standard errors.

To examine effects of lifetime motherhood, we computed the prevalence of alcohol use disorders among women who had ever been mothers and women who had never been mothers and then compared “Black” and “White” women. In order to examine the role of psychological measures, associations between the predictors and AUD were estimated using logistic regressions. All continuous predictors were standardized to the entire NESARC population mean.

## 3. Results

The demographic characteristics of the sample are presented in Table 1. On average, White women were slightly older than the Black women. A larger proportion of Black than White women had migrated to the US. Almost half of the sample reported a personal income below 20,000 USD. Similar proportions of Black and White women reported ever having children suggesting that attaining motherhood was not skewed by race.

**Figure 2:**
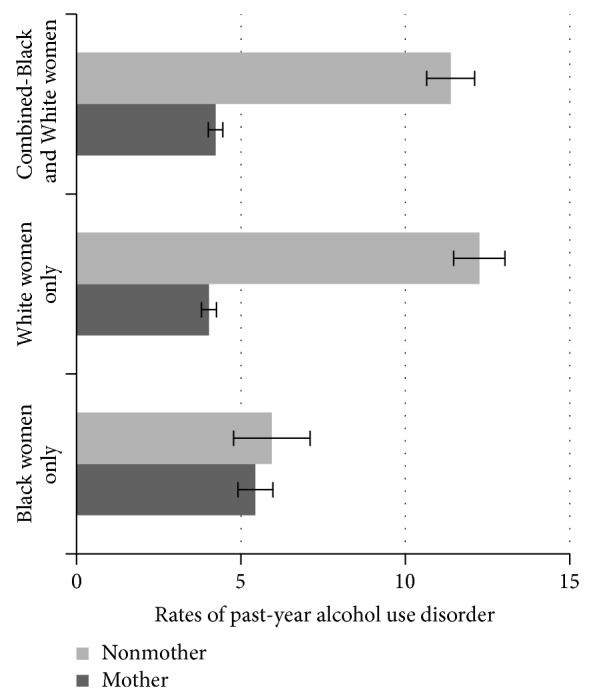
Prevalence of alcohol use disorders in the NESARC subsamples of Black and White women. Weighted proportions are presented.

The prevalence of alcohol use disorder (see Figure 2) was lower among mothers compared to nonmothers. However, when the prevalence of disorders was examined by race, different trends were observed. A motherhood advantage was observed for White women but not Black women.

Table 2 lists psychological characteristics. Family history of alcoholism was greater among both Black and White mothers as compared to nonmothers. Curiously, mothers reported lower discrimination than did nonmothers. Black women reported slightly elevated levels of perceived stress but smaller social networks and less social support. Black women also reported networks that are more racially mixed than White women and low levels of racial identification.

Finally, we performed logistic regressions to examine the associations of psychological risks and resources with the presence of an AUD.  Table 3a presents the bivariate associations of the predictors and AUD. As expected, family history of alcoholism predicted an AUD. However, this association was significant for White but not Black women. Several psychological risks and resources also predicted AUD including perceived stress and racial networks.

When all psychological risks and resources were included simultaneously and adjusted for demographic characteristics some differences in associations occurred between Black and White women by parenthood status (see Table 3b). Significantly, the associations between racial networks and AUD were seen only for Black women with children. Intriguingly, a similar association was also noted for White women without children. Perceived discrimination was associated with AUD among both White and Black women with children. No such associations were observed for women without children.

## 4. Discussion

In this study we examined motherhood effects on AUD, potential psychological mechanisms, and racial disparities. Using data from a large US national survey, we examined whether lifetime motherhood presented an advantage for current AUD for both Black and White women alike. Subsequently, we examined the role of differential psychological risks and resources for AUD at each race-motherhood intersection. Overall rates of AUD suggest a general motherhood advantage; that is, mothers reported lower AUD than nonmothers. However, when the racial groups were examined separately, motherhood advantage was only observed for White women. This is consistent with the notion that life-role-related AUD may be different for Black women [12–14, 19, 23–25]. This also calls for developing a better understanding of family and life role processes that may be affecting AUD specifically for Black women and begs for further exploration among other racial and ethnic minorities.

Black women with no children (i.e., nonmothers) had lower rates of AUD compared to White nonmothers. Extending this finding, we speculate that Black mothers with AUD are developing AUD at a later age, yet the underlying processes and reasons for this process are not entirely clear. Underlying race specific patterns in both motherhood processes and AUD onset and progression need to be understood and leveraged to allow for the development of culturally sensitive and effective treatments for AUD. Furthermore, if motherhood advantage for White women is due to the availability of screening and treatment when White women become mothers, then Black women may not be receiving adequate screening at the time that they become mothers. Perceived discrimination further increased the odds of AUD among mothers. We speculate that these multiple factors cumulatively increase disadvantage especially among Black mothers. Potential areas in which women and especially mothers experience discrimination need to be identified to allow for the development of better treatment for women with AUD [10, 19, 21].

An additional race specific pattern that we observed related to the associations of family history of alcohol problems with current AUD. Family history was associated with past-year AUD only for White women among both mothers and nonmothers alike. Psychological predictors were more significant in explaining AUD among Black women. Notable among these were perceived discrimination, perceived stress, and mixed race-ethnic networks. Mixed race-ethnic networks were protective for AUD among Black mothers. Perhaps women with mixed race-ethnic networks also have access to many social and economic benefits available to advantaged groups. Conversely, Black mothers whose networks were racially isolated were disadvantaged [35, 36]. This is consistent with the literature that social segregation may lead to a higher level of drinking as a form of coping with social stressors [36]. Quite surprisingly, we found a similar effect of mixed racial networks for White nonmothers. This is consistent with the literature suggesting that women's drinking is sensitive to peer group cultural norms [9]. Perhaps White women who were associated with other White women (where norms for drinking were supportive) had higher levels of AUD. This is also consistent with the sociological literature on friendship segregation in schools [32], where segregation has been found to be beneficial to racial groups whose drinking norms disfavored drinking.

Higher perceived stress increased the odds of an AUD across both racial groups and among mothers and nonmothers alike. Therapies like mindfulness training could be combined with AUD treatment to reduce perceived stress. An important negative finding pertains to social support which was not independently associated with AUD. This is, however, consistent with some findings that social support has little effect on problem drinking when another social disadvantage is present [30].

Overall, our findings highlight the significance of disentangling life roles at the intersection of race among women. Even though AUD is a medical condition, social etiology and intersecting risks from race and motherhood are significant and important. Future studies should disentangle and explore these processes in greater detail.

*Limitations*. Our findings need to be interpreted in the context of several limitations. First of all, because wave 1 of the national survey did not contain psychological measures, our data were cross-sectional, and causality cannot be inferred. Rather, our study points to differential life role processes among Black and White women [23, 24] and extends this to motherhood. Additional community-based studies using prospective study designs are needed to fully comprehend the underlying reasons for why motherhood advantage accrues only to White women.

Second, our study was based on secondary data analysis of publicly available limited use data and community level measures could not be included. Third, most results were based on self-reported data, which may be subject to recall bias. No physiological measures pertaining to perceived stress and AUD were available. Fourth, our measures primarily focused on differential aspects of social context; thus race specific metabolic and genetic factors were not considered. Fifth, the biological relationship of mothers with their children is not known and could not be considered.

Despite these limitations, the study's findings contribute to the growing literature on health disparities by extending a focus to AUD. In particular, AUD among women has received less attention relative to other health conditions. We highlighted heterogeneity in psychological processes underlying AUD for Black and White women. Future studies and prevention programs must consider these racial differences critically.

## 5. Conclusion

Using cross-sectional national survey data, we examined whether the notion of motherhood advantage for alcohol use disorders (AUD) was applicable to both Black and White women. This advantage did not accrue to Black women. We found that intersecting psychological risks and resources predicted a current AUD among Black and White mothers and nonmothers. Perceived stress was associated with AUD for all women. Discrimination and racial-ethnic networks were significant for Black mothers. Psychological factors had stronger effects on AUD than family history of AUD for Black women but not White women. Future studies should critically consider mechanisms by which race, motherhood, and psychological factors interactively affect AUD in women.

## Figures and Tables

**Table 1:** Demographic characteristics of the NESARC subsample of Black and White women.

		Combined	Black women	White women
		(*N* = 15150)	(*N* = 4133)	(*N* = 11017)
		% (SE) or mean	% (SE) or mean	% (SE) or mean
Age	20–90	49.78 (0.21)	45.74 (0.33)	50.46 (0.24)
Nativity	Immigrated	5.25 (0.71)	9.49 (2.27)	4.53 (0.51)
Marital status	Married	60.04 (0.62)	36.06 (0.92)	64.07 (0.58)
Employment	Employed (more than 35 hours per week)	57.60 (0.54)	50.59 (1.01)	58.78 (0.60)
Income	Less than 20,000 USD	53.72 (0.75)	54.04 (1.39)	53.66 (0.77)
Parenthood	Yes	80 (0.55)	80.95 (1.07)	79.84 (0.56)

Weighted proportions are presented.

**Table:** (a) Categorical predictors of interest

	Combined		Black women		White women	
	Nonmothers	Mothers	Nonmothers	Mothers	Nonmothers	Mothers
	% (SE)	% (SE)	% (SE)	% (SE)	% (SE)	% (SE)
Family history of alcohol problems	27.89 (1.06)	37.15 (0.66)	24.86 (2.01)	37.55 (0.68)	28.37 (1.07)	34.84 (1.13)
Perceived discrimination	8.82 (0.60)	4.74 (0.23)	10.70 (1.62)	4.26 (0.26)	8.52 (0.65)	7.58 (0.52)

Weighted prevalence is presented.

**Table:** (b) Continuous predictors of interest

		Combined		Black women		White women	
		Nonmothers	Mothers	Nonmothers	Mothers	Nonmothers	Mothers
		Mean (SE)	Mean (SE)	Mean (SE)	Mean (SE)	Mean (SE)	Mean (SE)
Perceived stress	High = greater stress	1.99 (0.01)	2.03 (0.01)	2.15 (0.03)	2.17 (0.02)	1.97 (0.02)	2.01 (0.01)
Social support networks	High = greater support	8.89 (0.02)	8.80 (0.01)	8.78 (0.04)	8.74 (0.02)	8.90 (0.02)	8.80 (0.01)
Perceived social support	High = more perceived social support	3.58 (0.01)	3.56 (0.01)	3.53 (0.02)	3.51 (0.01)	3.58 (0.01)	3.56 (0.01)
Mixed racial-ethnic networks	High = less segregated racial-ethnic networks	6.59 (0.06)	6.35 (0.04)	7.31 (0.14)	7.22 (0.07)	6.48 (0.06)	6.21 (0.04)
Racial-ethnic identification	High = low identification with one's race ethnicity	2.45 (0.02)	2.31 (0.01)	2.15 (0.04)	2.07 (0.02)	2.50 (0.02)	2.35 (0.01)

Weighted means are presented.

**Table:** (a) Odds ratios (based on bivariate associations)

	Description	Black women		White women	
		Nonmothers	Mothers	Nonmothers	Mothers
		*N* = 750	*N* = 3383	*N* = 2334	*N* = 8683
		OR (CI)	OR (CI)	OR (CI)	OR (CI)
Family history	No alcoholism	0.72 (0.32–1.63)	0.73 (0.49–1.08)	0.56 (0.42–0.74)∗	0.42 (0.33–0.53)∗
Perceived discrimination	No discrimination	0.35 (0.14–0.84)∗	0.28 (0.17–0.46)∗	0.46 (0.30–0.71)∗	0.19 (0.13–0.26)∗
Perceived stress	High = greater stress	1.28 (1.10–1.50)∗	1.19 (1.09–1.31)∗	1.21 (1.13–1.29)∗	1.20 (1.14–1.27)∗
Social support networks	High = greater support	1.30 (1.05–1.59)∗	1.26 (1.12–1.41)∗	1.18 (1.10–1.28)∗	1.14 (1.05–1.24)∗
Perceived social support	High = more perceived social support	0.95 (0.82–1.11)	0.96 (0.88–1.04)	1.04 (0.97–1.10)	0.93 (0.88–0.98)∗
Mixed racial-ethnic networks	High = less segregated racial-ethnic networks	1.01 (0.84–1.20)	0.87 (0.77–0.99)∗	0.99 (0.91–1.08)	1.06 (0.99–1.14)
Racial-ethnic identification	High = low identification with one's own race ethnicity	0.95 (0.65–1.32)	1.07 (0.81–1.40)	1.11 (0.97–1.26)	1.15 (1.01–1.32)

^*^ *P* < 0.05; OR: odds ratios; CI: confidence intervals.

(1) All continuous predictors are standardized to the mean.

**Table:** (b) Adjusted odds ratios

	Description	Black women		White women	
		Nonmothers	Mothers	Nonmothers	Mothers
		*N* = 750	*N* = 3383	*N* = 2334	*N* = 8683
		OR (CI)	OR (CI)	OR (CI)	OR (CI)
Age	Continuous	0.73 (0.55–0.98)∗	0.72 (0.62–0.83)∗	0.65 (0.57–0.73)∗	0.66 (0.60–0.53)∗
Marital status	Married	0.91 (0.25–3.40)	0.86 (0.52–1.41)	0.53 (0.34–0.84)∗	0.71 (0.53–0.95)∗
Employment	Employed less than 35 hours per week	0.68 (0.25–1.87)	0.99 (0.61–1.60)	0.88 (0.60–1.28)	0.72 (0.54–0.96)∗
Income	More than 20000 USD	1.25 (0.47–3.33)	1.17 (0.71–1.94)	1.28 (0.89–1.84)	1.27 (0.93–1.74)
Nativity	Born in the United States	7.47 (0.93–60.14)	1.17 (0.50–2.75)	1.95 (0.77–4.91)	2.14 (0.95–4.79)
Family history	No alcoholism	0.75 (0.32–1.77)	0.8 (0.55–1.15)	0.54 (0.40–0.72)∗	0.52 (0.40–0.66)∗
Perceived discrimination	No discrimination	0.45 (0.18–1.10)	0.4 (0.24–0.65)∗	0.75 (0.48–1.16)	0.34 (0.24–0.49)∗
Perceived stress	High = greater stress	1.27 (1.05–1.53)∗	1.18 (1.08–1.29)∗	1.24 (1.14–1.34)∗	1.11 (1.04–1.18)∗
Social support networks	High = greater support	1.19 (0.76–1.87)	1.12 (0.94–1.32)	1.11 (0.94–1.32)	0.96 (0.88–1.05)
Perceived social support	High = more perceived social support	0.99 (0.79–1.23)	1 (0.93–1.08)	1.02 (0.94–1.11)	0.96 (0.90–1.02)
Mixed racial-ethnic networks	High = less segregated racial ethnic networks	1.05 (0.86–1.30)	0.81 (0.70–0.94)∗	0.90 (0.82–1.00)∗	0.97 (0.89–1.05)
Racial-ethnic identification	High = low identification with one's own race ethnicity	0.83 (0.55–1.24)	1.07 (0.77–1.48)	1.11 (0.95–1.30)	1.01 (0.86–1.20)

^*^ *P* < 0.05; OR: odds ratios; CI: confidence intervals.

(1) All continuous predictors are standardized to the mean.
